# A pre-pandemic COVID-19 assessment of the costs of prevention and control interventions for healthcare associated infections in medical and surgical wards in Québec

**DOI:** 10.1186/s13756-021-01000-y

**Published:** 2021-10-21

**Authors:** Eric Tchouaket Nguemeleu, Stephanie Robins, Sandra Boivin, Drissa Sia, Kelley Kilpatrick, Bruno Dubreuil, Catherine Larouche, Natasha Parisien, Josiane Letourneau

**Affiliations:** 1 Department of Nursing Research, Université du Québec en Outaouais, St-Jérôme Campus, 5, rue Saint-Joseph, Office J-2204, Saint-Jérôme, Québec J7Z 0B7 Canada; 2 Centre Intégré de Santé et de Services Sociaux Des Laurentides, Direction de la Santé Publique, Saint-Jérôme, Québec Canada; 3 Ingram School of Nursing, McGill University, Montreal, Québec Canada; 4 Centre Intégré de Santé et de Services Sociaux de Laval, Laval, Québec Canada; 5 Centre Intégré Universitaire de Santé et de Services Sociaux du Saguenay–Lac-Saint-Jean, Laval, Canada; 6 Institut National de Santé Publique du Québec, Quebec City, Québec Canada

**Keywords:** Healthcare associated infection, Prevention and control, Clinical best practice, Cost, Time-driven activity-based costing, Time-motion study, COVID-19

## Abstract

**Background:**

Healthcare-associated infections (HCAIs) present a major public health problem that significantly affects patients, health care providers and the entire healthcare system. Infection prevention and control programs limit HCAIs and are an indispensable component of patient and healthcare worker safety. The clinical best practices (CBPs) of handwashing, screening, hygiene and sanitation of surfaces and equipment, and basic and additional precautions (e.g., isolation, and donning and removing personal protective equipment) are keystones of infection prevention and control (IPC). There is a lack of rigorous IPC economic evaluations demonstrating the cost–benefit of IPC programs in general, and a lack of assessment of the value of investing in CBPs more specifically.

**Objective:**

This study aims to assess overall costs associated with each of the four CBPs.

**Methods:**

Across two Quebec hospitals, 48 healthcare workers were observed for two hours each shift, for two consecutive weeks. A modified time-driven activity-based costing framework method was used to capture all human resources (time) and materials (e.g. masks, cloths, disinfectants) required for each clinical best practice. Using a hospital perspective with a time horizon of one year, median costs per CBP per hour, as well as the cost per action, were calculated and reported in 2018 Canadian dollars ($). Sensitivity analyses were performed.

**Results:**

A total of 1831 actions were recorded. The median cost of hand hygiene (N = 867) was 20 cents per action. For cleaning and disinfection of surfaces (N = 102), the cost was 21 cents per action, while cleaning of small equipment (N = 85) was 25 cents per action. Additional precautions median cost was $4.1 per action. The donning or removing or personal protective equipment (N = 720) cost was 76 cents per action. Finally, the total median costs for the five categories of clinical best practiced assessed were 27 cents per action.

**Conclusions:**

The costs of clinical best practices were low, from 20 cents to $4.1 per action. This study provides evidence based arguments with which to support the allocation of resources to infection prevention and control practices that directly affect the safety of patients, healthcare workers and the public. Further research of costing clinical best care practices is warranted.

**Supplementary Information:**

The online version contains supplementary material available at 10.1186/s13756-021-01000-y.

## Introduction

Healthcare-associated infections (HCAIs) present a major public health problem that significantly affects patients, health care providers and the entire healthcare system. After necessary care in a clinic, hospital or long-term care facility, these infections can arise at surgical sites, following antibiotic therapy, or occur due to the use of devices such as ventilators, implants or catheters [1]. Recent point prevalence surveys establish a range of HCAI rates across low, middle and higher income countries [2, 3]. The highest rates of infection—up to 51.8%—occur in intensive care units, whereas overall hospital infection rates vary from 2.9% in six hospitals in Greece, to 9.2% in Australia to 10.4% in 25 hospitals in Canada and 14.3% in two hospitals in Ethiopia. Since most of these infections are considered preventable [4], they are seen as an indicator of the quality of patient care and safety. HCAIs untowardly affect patients and their caregivers as they result in medical complications, high rates of morbidity and mortality, and reduced quality of life [5]. They also burden healthcare systems in extra costs related to the prolonged length of stay or readmission of patients, patient’s care-related expenses and costs involved in limiting further contagion [6, 7].

Accordingly, infection prevention and control (IPC) is an indispensable component of patient and healthcare worker safety [8]. Essential IPC measures include four transverse clinical best care practices (CBPs) that apply across all care settings: a) hand hygiene; b) hygiene and sanitation including the cleaning and disinfecting of equipment and surfaces; c) application of basic and additional precautions (e.g., isolation, and donning and removing personal protective equipment) and; d) screening of carriers and patients who are at risk [9]. These established components of IPC have been validated by extensive clinical practice and are incorporated in IPC guidelines set forth by the World Health Organization (WHO)[8], Health Canada and the Canadian Patient Safety Institute [10, 11] as well as the US Centers for Disease Control and Prevention (CDC) [12]. In the current COVID-19 pandemic, these CBPs have proven vital in reducing the spread of infection in healthcare facilities [13, 14].

IPC programs that incorporate CBPs have been shown to be clinically, and from an institutional and government standpoint, cost effective [15, 16]. Despite this, only a small proportion of most healthcare budgets are dedicated to public health activities and management costs, which generally include IPC costs. In 2019, the Canadian Institute for Health Information estimated Canada would spend $264.4 billion Canadian dollars on health care, with 8.6% or 602 million used for public health activities and administration costs [17]. In Quebec’s Economic Plan of 2018, only 10.7% of the healthcare budget (representing $2.3 billion of $21.8 billion) was destined for general management, public health activities and administration costs [18]. In general, IPC is only a small part of these three sections of the budget. A central hypothesis suggests this lack of investment is due to the dearth of rigorous empirical evidence demonstrating the cost–benefit of IPC programs. Systematic reviews of IPC economic evaluations report this gap in research in general, and point to the lack of assessment of the value of investing in CBPs more specifically [6, 15, 19].

Comprehensive costing of activities involved in IPC must consider the costs of human resources and the costs of materials and products used. Analyzing human resource expenses can be undertaken using time-driven activity-based costing (TDABC), a process-oriented cost-accounting methodology increasingly used in healthcare to measure human resource costs [20, 21]. TDABC functions by dynamically allocating expenses related to the consumption of resources across human-driven processes, with the purpose of summing them throughout the care delivery value chain [22]. We previously developed a time and motion guide that captures resources consumed by healthcare workers (HCWs) [23]. The guide was specifically developed to assess the costs required to perform the CBPs presented above. It captures: (i) the time healthcare staff spend on: hand hygiene, cleaning and disinfecting healthcare equipment and the environment, donning and removing personal protective equipment (PPE), screening, basic practice and additional precautions (contact, droplet-contact or airborne), education, training and awareness campaigns; (ii) the materials used for these CBPs and; (iii) the products these CBPs require. Using micro-costing data collection that allows for cost estimates from the bottom up [24], the goal of this tool is to provide accurate IPC cost data to be used in economic healthcare evaluations and inform decision making [25].

As a first step in determining the cost–benefit of CBP IPC practices, this study aims to evaluate overall costs associated with each of the four CBPs by testing our time and motion guide in two healthcare facilities. To our knowledge, no study has undertaken an assessment of costs related to these four transverse CBPs, and none using a systematic and validated time and motion instrument. Moreover, no studies have been undertaken in Canada. Here we present the measurement of CBP costs in two healthcare facilities in the province of Québec, Canada. This evaluation will provide real cost data for healthcare decision makers regarding IPC implementation and optimal clinical practices. It will also provide researchers with insight about pre pandemic IPC healthcare costs, which will serve as a baseline from which to compare actual COVID-19 pandemic costs of IPC measures that were instituted in March of 2020.

### Conceptual framework and research questions

**Fig. 1:**
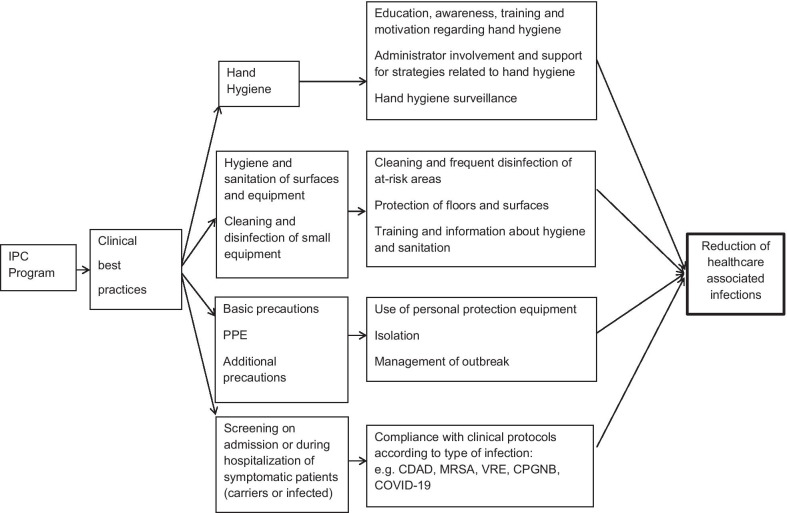
Infection prevention and control clinical best practices

Our conceptual IPC framework is presented in Fig. 1, where the four CBPs are outlined. This framework was adapted from the theoretical framework of Resar and colleagues [9], and previously used by our team [23]. In this study, we aim to answer the following questions in the context of an acute care setting: what are the human and material resource costs of: 1) hand hygiene, 2) hygiene and sanitation practices, and disinfection of equipment, 3) basic practices and additional precautions (donning and removing personal protective equipment or PPE) and 4) screening?

## Methods

### Design

A prospective observational study was used. Data were collected in the pre-COVID-19 period between November 28^th^ and December 15^th^, 2018 from one hospital in the Saguenay-Lac-Saint-Jean region and one hospital in the Laurentian region of Québec, Canada. We chose a university and non-university based integrated health and social services centre.

### Participants and procedures

HCWs were selected from acute-care wards of medicine and surgery as these wards see and treat the highest number of hospitalized patients. In each hospital, the selected staff included 6 nurses, 6 auxiliary nurses, 6 orderlies and 6 hygiene and sanitation workers providing a final sample of 48 (24 from each site). The nurses (N = 12) had on average 8.5 years’ experience in the position, auxiliary nurses 14.3, orderlies had 7.1 and hygiene and sanitation workers 7.3 years respectively. The HCWs observed had a wide range of experience, from less than one year of experience to very experienced (more than 20 years). Prospective participants were approached by the research team and unit managers, or at information sessions, and had the study explained to them. If they agreed, participants provided written informed consent. Each participant was directly observed by a nurse researcher over a two-hour period during their regularly scheduled work shift (day or evening), for two consecutive weeks. The time spent in relation to each CBP (hand hygiene; hygiene and sanitation including the cleaning and disinfecting of equipment and surfaces; application of basic and additional precautions including donning and removing of PPE and screening of carriers and patients who are at risk), along with all materials and product used were measured. Ethical approval was obtained from the sites where recruitment took place (study # MP-28–2018-002).

### Time and motion guide

The development of the IPC time and motion guide used in this study has been previously described [23]. It was based on an algorithm developed by our team, and published. Validated by Delphi review, the guide contains eight dimensions of human and material resource assessment. These include: Identification [83 items]; Personnel [5 items]; Additional Precautions [1 item]; Hand Hygiene [2 items]; Personal Protective Equipment [14 items]; Screening [4 items]; Cleaning and Disinfection of Patient Care Equipment [33 items]; and Hygiene and Sanitation [24 items]. Observers follow one HCW and, using an online version of the guide, systematically record time of actions as triggered from the beginning to the end of the action, using chronometric measurement. The observer inputs products (e.g. hand soap or hydroalcoholic solution) and disposable and reusable materials (e.g. gowns, wipes, bedpans) used during these procedures. We followed the suggested time and motion definitions set out by Lopetegui et al*.* [26]. As these authors suggest, for time measurement, the guide requires an ‘external observer capturing data continuously’ and is characterized by a ‘milestone’ study schema, where all work time in relation to a particular action (motion) is measured in seconds. This process allows for the collection of valid and objective real-time measurements (Additional files 1 and 2).

### The procedure of data analysis for costing

**Fig. 2:**
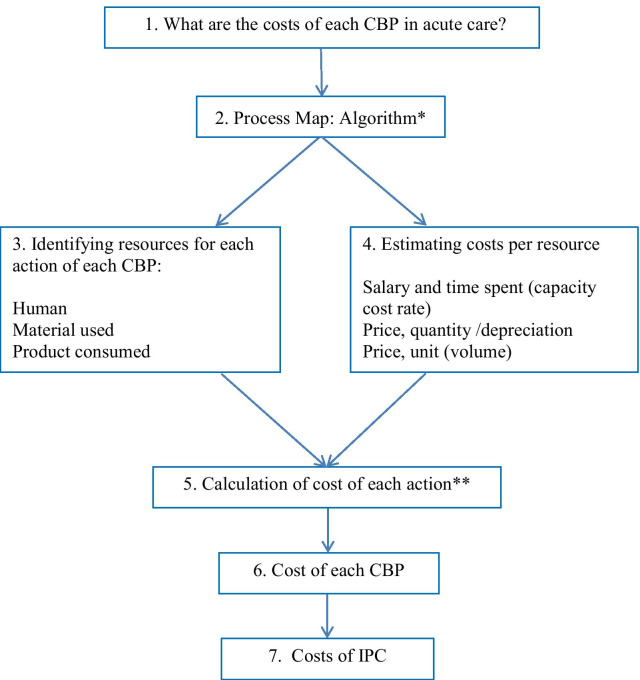
Costing procedure framework. *See Additional file 1. **Cost calculations are described in Table 1

We used a hospital perspective with a time horizon of one year as reported in 2018. All costs were actualized to 2021 using a discount rate of 3%, 5% and 8% and reported in Canadian dollars ($ CAD). All human resource costs (salaries) were based on standardized government salaries for each HCW [27]. Costs of all supplies were obtained from a standardized provincial public healthcare pricelist for hospitals 2017–2018 [28] (see Additional file 3). Our costing procedure (see Fig. 2) is similar to that described by da Silva Etges et al.[29], who propose a modified 8-step TDABC micro-costing framework originally set out by Keel and colleagues [21, 29]. These authors break down healthcare-based costing into: 1) defining the study question or process to be assessed; 2) mapping the process; 3) identifying the resource groups (structure and personnel) used in each activity; 4) estimating the total cost of each resource group; 5) estimating capacity per resource and the cost capacity rate; 6) analysing the time required for each activity used during the process; 7) calculating the total cost of patient care and; 8) performing cost analytics (benchmarking, costing per phase of care etc.). We followed the above in our costing process, with several small adaptations. First, we defined our process not as care delivery as seen from the patient perspective, but rather as those events undertaken by HCWs in relation to CBPs performed across a set period of time. Second, our resource groups did not include overhead costs, all of our costs were operating expenses (here defined as human, material and product resources used in IPC). Finally, we simplified our capacity cost rate [30] for human resource costing: our numerator was the worker’s salary and their benefits, the denominator was an hour of time.

### Costing

To obtain human resource costs, the number of actions undertaken by HCWs was multiplied by the average time (in seconds) required for that action and by the average salary (including benefits) for that HCW category. To calculate material costs, the total number of disposable items (e.g. microfiber cloths, masks) that were used was multiplied by the price per item. For materials that were reusable, the total number of items used was multiplied by the depreciation rate of that item (i.e. number of possible uses). Products were costed by multiplying the number of actions by the cost per volume of product used for each action (see Table 1). These calculations provided a total cost over the two-hour observation period. We also report cost per hour. IBM SPSS Statistics for Mac, Version 26.0 (Armonk, NY: IBM Corp) was used for all calculations of cost.

**Table 1:** Cost calculation of clinical best practice actions

Resource Cost	Unit	Multiplied by
Human	All time in seconds converted to hourly scale	Hourly wage including benefits
Material: disposable	Quantity used	Price per item
Material: reusable	Quantity used	% depreciation (number of times used)
Product (e.g. soap)	Millilitres (mls) of product	Price per item
Product (e.g. disinfectant)	Millilitres (mls) of diluent	Price per ml*
Total Costs of CBPs	All human resource + material + product costs	

^*^Costs will be multiplied by proportion of total solution used or discarded

### Sensitivity analyses

The purpose of our sensitivity analysis was to estimate the robustness of the results by examining the extent to which they may have been affected by variation in IPC activities including: salaries based on healthcare workers experience, the time spent and volumes of product used. A sensitivity analysis also takes into account the value of unmeasured variables or assumptions [31]. In this study, for all human resources, sensitivity analyses were performed to account for the range of salaries (minimum to maximum) of professionals, depending on the salary scale for each profession. Sensitivity analyses were also performed for time spent performing each CBP using the minimum and maximum limit respectively. For products where the volume varies from a standard quantity depending on the user (e.g**.** millilitres of soap for hand washing), sensitivity analyses were performed by using a range of volumes. Using Monte Carlo simulation, we varied each cost over 1,000 iterations, to obtain 2.5% and 97.5% percentiles and estimate the 95% confidence interval. Microsoft excel was used to perform the sensitivity analyses.

### Comparison analyses

We compared time spent across category (e.g. human resource or product) within each CBP using a 5% threshold and non-parametric tests (Mann–Whitney Wilcoxon, Kruskal Wallis) [32].

## Results

### Times and actions used for clinical best practices

All CBP times are described in Table 2.

**Table 2:** Number of actions and time used for clinical best practices

Clinical best practice	Actions		Median time (seconds)	Percentiles		Mean time (seconds)	S.D. (seconds)	Minimum Time (seconds)	Maximum time (seconds)	Kruskal Wallis		
	N	%		2.5	97.5					X^2^	df	P
Hand hygiene	867	100	14.3	4.0	35.7	15.6	8.3	1.4	109.9			
Healthcare personnel												
Nurse: bachelor trained	21	2.4	14.9	11.0	27.9	16.9	5.1	11.0	27.9	21.27	4	0.000
Nurse: college trained	125	14.4	16.0	4.2	40.0	17.8	11.5	2.5	109.9			
Auxiliary nurse	244	28.1	13.5	3.2	38.5	15.2	8.7	2.4	49.3			
Orderly	347	40.0	14.6	5.6	35.8	15.8	7.2	2.7	46.7			
Hygiene and sanitation staff	130	15.0	12.4	3.0	31.0	13.5	6.6	1.4	41.5			
Moment												
Before patient contact	193	22.3	14.1	5.1	34.3	15.0	7.0	2.6	40.2	36.86	4	0.000
Before aseptic procedure	17	2.0	21.5	5.2	32.5	20.1	7.1	5.2	32.5			
After patient contact	601	69.3	14.1	3.4	32.7	15.0	7.3	1.4	49.3			
After body fluid exposure	37	4.3	14.9	4.0	110.0	19.9	18.6	4.0	110.0			
Other	19	2.2	24.1	14.6	43.4	27.6	9.7	14.6	43.4			
Products used												
Soap and water	71	8.2	22.2	8.5	44.1	23.5	10.0	8.2	46.7	54.18	2	0.000
Hydroalcoholic solution wall	761	87.8	14.0	4.0	32.7	14.9	7.8	1.4	109.9			
Hydroalcoholic solution table (500 ml)	32	3.7	13.8	2.6	29.4	13.8	7.7	2.6	29.4			
Hydroalcoholic solution pocket size (45–50 ml)	3	0.3	27.8	27.3	36.4	30.5	5.1	27.2	36.4			
Hygiene and sanitation of surfaces	102	100	541.5	42.9	1840.2	598.6	486.1	35.4	3411.6			
Healthcare personnel												
Hygiene and sanitation staff	102	100	541.5	42.9	1840.2	598.6	486.1	35.4	3411.6	n/a		
Kind of cleaning												
Daily regular	58	56.9	333.6	41.5	1140.6	432.7	300.1	35.4	1222.2	32.45	3	0.000
Daily additional precautions	13	12.7	335.9	51.4	702.0	391.2	181.6	51.4	702.0			
Terminal	3	2.9	738.6	35.8	3411.6	1395.3	1781.1	35.8	3411.6			
Terminal discharge/transfer	28	27.5	872.5	406.0	1941.0	953.1	404.5	406.0	1941.0			
Materials used*												
Reusable cloths	6	5.9	635.9	450.7	1492.8	753.0	390.2	450.7	1492.8	n/a		
Microfibre-reuseable	97	95.1	554.9	41.4	1862.1	611.7	493.6	35.4	3411.6			
Disinfectant disposable wipes	1	1.0	565.5	565.5	565.5	565.5	–	565.5	565.5			
Alcohol swabs	0	0	–	–	–	–	–	–	–			
Floor buffers	19	18.2	596.8	320.4	1531.7	715.2	350.1	320.4	1531.7			
Mops	1	1.0	58.6	58.6	58.6	58.6	–	58.6	58.6			
Toilette brush	1	1.0	151.9	151.0	151.9	151.9	–	151.9	151.9			
Cleaning of small equipment	85	100	31.3	10.8	91.3	34.8	18.6	8.7	94.9			
Healthcare personnel												
Nurse: bachelor trained	2	2.4	21.8	21.1	22.6	21.8	1.1	21.1	22.6	4.15	4	0.386
Nurse: college trained	23	27.1	30.0	11.7	57.2	33.5	12.7	11.7	57.2			
Auxiliary nurse	38	44.7	28.0	8.7	94.9	35.3	23.7	8.7	94.9			
Orderly	12	14.1	36.1	14.0	85.7	39.4	17.7	14.0	85.7			
Hygiene and sanitation staff	10	11.8	36.0	16.6	42.6	33.1	8.6	16.6	42.6			
Type of small equipment												
Multifunction vital sign monitor	29	34.1	39.7	11.5	94.9	43.3	20.8	11.5	94.9			
Blood glucose monitor	18	21.2	20.7	8.7	51.7	21.6	10.0	8.7	51.7			
Chariot and mop handle	10	11.8	32.2	16.6	42.6	30.5	9.0	16.6	42.6			
Positive displacement pump	4	4.7	58.3	40.9	83.2	60.2	17.4	40.9	83.2			
Pulse oximeter	4	4.7	37.0	21.1	65.0	40.0	18.7	21.1	65.0			
Portable bladder scanner	3	3.5	21.9	20.8	23.0	21.9	1.1	20.8	23.0			
Wheelchair	3	3.5	22.1	14.0	34.7	23.6	10.5	14.0	34.7			
Commode chair	3	3.5	32.5	25.7	44.6	34.3	9.5	25.7	44.6			
Walker	2	2.4	36.0	20.2	51.8	36.0	22.4	20.2	51.8			
Scissors	3	3.5	15.4	11.7	29.2	18.7	9.2	11.7	29.2			
Emesis bowl	3	3.5	37.5	29.9	42.0	36.4	6.1	29.9	42.0			
Thermometer	2	2.4	80.0	65.0	94.9	80.0	21.2	65.0	94.9			
Stretcher	1	1.2	85.7	85.7	85.7	85.7	–	85.7	85.7			
Laundry bag support	1	1.2	44.7	44.7	44.7	44.7	–	44.7	44.7			
Bassin	1	1.2	35.7	35.7	35.7	35.7	–	35.7	35.7			
Scale	1	1.2	29.3	29.3	29.3	29.3	–	29.3	29.3			
Doppler	1	1.2	37.3	37.3	37.3	37.3	–	37.3	37.3			
Table	1	1.2	33.3	33.3	33.3	33.3	–	33.3	33.3			
Comb	1	1.2	22.4	22.4	22.4	22.4	22.4	22.4	22.4			
Other	18	21.2	33.2	11.7	44.7	31.8	9.2	11.7	44.7			
Additional precautions	57	100	274.3	16.1	2669.0	385.0	511.7	15.5	3445.8			
Healthcare personnel												
Nurse: bachelor trained	5	8.8	169.1	32.3	205.4	138.9	77.4	32.3	205.4	23.09	3	0.000
Nurse: college trained	0	0	–	–	–	–	–	–	–			
Auxiliary nurse	17	29.8	199.8	15.5	579.9	247.8	160.9	15.5	579.9			
Orderly	16	28.1	117.6	16.8	786.5	197.5	213.0	16.8	786.5			
Hygiene and sanitation staff	19	33.3	542.8	89.8	3445.8	730.3	748.7	89.8	3445.8			
Kind of additional precaution												
Contact	42	73.7	252.9	16.7	3316.4	427.7	586.0	15.5	3445.8	4.96	3	0.000
Contact-droplet	12	21.1	340.9	89.8	463.8	313.4	114.3	89.8	463.8			
Droplet	2	3.5	100.5	84.9	116.1	100.5	22.1	84.9	116.1			
Protection (inversed)	1	1.8	16.8	16.8	16.8	16.8	–	16.8	16.8			
Airborne	–	–	–	–	–	–	–	–	–			
Airborne-contact	–	–	–	–	–	–	–	–	–			
Personal Protective Equipment (PPE)	720	100	11.6	2.8	48.1	14.0	10.6	0.3	94.4			
Healthcare personnel												
Nurse: bachelor trained	12	1.7	16.5	9.9	58.7	24.6	15.2	9.9	58.7	11.09	4	0.026
Nurse: college trained	37	5.1	11.1	3.9	28.3	12.1	5.9	3.9	28.3			
Auxiliary nurse	125	17.4	11.8	1.9	40.3	13.9	9.9	1.6	76.0			
Orderly	243	33.8	11.5	4.5	39.0	13.2	7.9	3.2	65.5			
Hygiene and sanitation	303	42.1	11.7	2.0	64.5	14.3	12.6	0.3	94.4			
PPE used												
Disposable gown	160	22.2	13.2	5.9	75.4	18.7	15.7	1.7	94.4	n/a		
Reusable gown	597	82.9	11.6	2.6	49.0	13.7	10.6	0.3	94.4			
Gloves—sterile (pair)	700	97.2	11.5	2.7	40.5	13.5	10.0	0.3	94.4			
Gloves—nitrile	718	99.7	11.6	2.8	47.4	13.9	10.4	0.3	94.4			
Gloves—vinyl	702	97.5	11.5	2.7	43.8	13.6	10.2	0.3	94.4			
Procedural or surgical mask	720	100.0	11.6	2.8	48.1	14.0	10.6	0.3	94.4			

^*^NB the number of materials surpasses the total number of actions as actions can use more than one material/product

SD = Standard deviation

X^2^ = Chi-square statistic

df = degrees of freedom

### Hand hygiene

A total of 867 hand hygiene actions took place; median hand washing time was 14.3 s (range 1.4—109.9) across all staff (nurses, orderlies and hygiene and sanitation staff). There was a significant difference X^2^ = 21.27 *p* = 0.000 between groups. Moreover, the moment when hands were washed differed significantly X^2^ = 36.86 *p* = 0.000 and was highest before an aseptic procedure at 21.5 s.

With regards to different products used, pocket size hydroalcoholic solution had the longest median hand washing time at 27.8 s, followed by soap and water at 22.2 s, then table sized hydroalcoholic solution 500 ml, and wall at 14.0 and 13.8 s respectively. Time across these products differed significantly X^2^ = 54.18 *p* = 0.000.

### Hygiene and sanitation of surfaces and cleaning and disinfecting of equipment

A total of 102 actions took place to clean surfaces, exclusively by hygiene and sanitation staff. The median time spent cleaning was 541.6 s (or 9 min and 2 s) range 35.4 s to 3411.6 s (56 min and 52 s). The most time was dedicated to terminal cleaning (cleaning following patient transfer or discharge) with a median of 872.5 s (or 14 min and 33 s). The kind of cleaning differed X^2^ = 32.45 *p* = 0.000, significantly by category.

There were 85 actions related to the cleaning of small equipment with the median time being just under half a minute at 31.3 s (range 8.7—94.9). There was no significant difference between personnel.

### Personal protective equipment (PPE)

Furthermore, a total of 720 actions were related to the donning or removing of personal protective equipment (PPE). Median time for PPE was 11.6 s (range 1.0–94.4). Bachelor trained nurses had the highest median at 16.5 s per action compared to an average of 11 s for all other personnel, who differed significantly X^2^ = 23.09 *p* = 0.026.

### Additional precautions

For the CBP of additional precautions 57 actions were recorded, with a median time of 274.3 s (4 min and 34 s), (range 15.5—3445.8 s or 57 min and 26 s). There was a significant difference between personnel X^2^ = 23.09 *p* = 0.000. Contact droplet precautions were the kind of precaution with the highest median at 340.9 s. There were no observations of airborne or airborne-contact precautions during the study.

### Screening

There were insufficient recordings of screening procedures (N = 3) to describe them.

### CBP costs

A summary of CBP costs are described in Table 3.

**Table 3:** Costs of clinical best practices over two hour observation period

Clinical best practice costs	Median	Confidence interval		Median	Confidence interval	
	($)	2.5	97.5	$	2.5	97.5
		Percentile ($)	Percentile ($)		Percentile ($)	Percentile ($)
	N = 867			N = 1		
Hand hygiene total cost	170.2	26.4	335.5	0.2	0.03**	0.4
Human resource cost	159.7	20.0	321.9	0.2	0.02**	0.4
Product cost	10.4	6.4	13.6	0.01**	0.01**	0.02
	N = 102	($)		N = 1	($)	
Hygiene and sanitation of surfaces total cost	21.9	13.3	30.9	0.2	0.1	0.3
Human resource cost	9.2	0.7	18.2	0.9	0.01**	0.2
Product cost	12.7	12.7	12.7	0.1	0.1	0.1
	N = 85	($)		N = 1	($)	
Cleaning of small equipment total cost	21.5	6.6	40.4	0.3	0.1	0.5
Human resource cost	21.5	6.6	40.4	0.3	0.1	0.5
Reusable materials *						
Products for disinfection*						
	N = 57	($)		N = 1	($)	
Additional precautions total cost	235.3	26.6	462.8	4.1	0.3	5.4
Human resource cost	235.3	26.6	462.8	4.13	0.3	5.4
	N = 720	($)		N = 1	($)	
Personal protective equipment (PPE) total cost	546.8	445.0	660.7	0.8	0.6	0.9
Human resource cost	114.1	12.4	228.0	0.2	0.02**	0.3
PPE material	432.7	432.7	432.7	0.6	0.6	0.6
	N = 1831	($)		N = 1	($)	
Total clinical best practice costs for two hours of observation	996.2	518.3	1530.6	0.5	0.3	0.8
Total clinical best practice costs per hour	498.1	259.2	765.3	0.3	0.1	0.4

^*^There were insufficient (missing) data

^**^For all results where values were below 0.05 we kept two decimal places to better represent the value

Overall, for two hours of observation, the median cost for 867 actions of hand hygiene was $170.2 (95% CI: 26.4—335.5), which represents 20 cents per action. For cleaning and disinfection of surfaces, the cost was $ 21.9 (95% CI: 13.3 − 30.9) or 21 cents per action, while cleaning of small equipment (85 actions) was $21.5 (95% CI: 6.6–40.4) for human resource costs, or 25 cents per action. Material costs were not captured in this category. Additional precautions median cost was $235.3 (95% CI: 26.6- 462.8) or $4.1 per action. The 720 actions of donning or removing PPE median cost was $546.8 (95% CI: 445.0 – 660.7) or 76 cents per action. Screening costs were not calculated as the number of samples taken for screening was too small (N = 3). Finally, the total median costs for the five categories of CBP assessed were $996.2 for two hours (95% CI: 518.3–1530.6) of observation or $498.1 per hour (95% CI: 259.2 − 765.3), which equalled 27 cents total median individual CBP action cost per hour.

## Discussion

In this pilot project of TDABC we assessed the costs of time, materials and products required to undertake essential clinical IPC practices by observing 48 healthcare workers in two Quebec hospitals. Our findings reveal that the costs of preventing the transmission of infection are remarkably low, even when the action is performed by the highest paid personnel (median cost being 27 cents per action). Weighed against the risk of infection and illness and subsequent monetary and human cost, this analysis supports the existing literature that describes the cost and cost–benefit of investing in resources that support compliance with IPC measures [15, 33–35]. This study also importantly provides an assessment of costs of infection prevention in a pre-pandemic context and thus serves as a baseline against which to compare future healthcare economic analyses.

When performed properly**,** hand hygiene is considered the single most important way to limit the spread of communicable diseases [36]. Subjectively, healthcare personnel may consider the time spent on hand hygiene as adequate, however the times recorded in this study did not reach WHO guidelines [36]. Indeed, in our observations, median time for hand washing ranged from 12.4 and 16.0 s across personnel, with a median cost of 20 cents per action. When using soap and water, the WHO suggests 40–60 s for an entire wash from the beginning to the end of the activity; we recorded median times of 22.2 s or 55% of the lower WHO limit. When using hydroalcoholic solutions that were wall or table mounted, median times were 14.0 and 13.8 s respectively, which also did not meet the WHO guidelines of 20–30 s minimum for rubbing using an alcohol-based formulation, although it came slightly closer at 69% of the lower limit. Compliant hand washing was observed among personnel that used pocket size gel, with a median time of 27.8 s that surpassed the minimum limit of 20 s by seven seconds, or 39%. This may be due to the ease and efficiency of using a portable hand gel that allows for disinfecting one’s hands during other tasks, such as talking with a patient, or traveling between rooms. Some evidence exists for installing dispensers close to their place of use to improve handwashing compliance [37]. However, a recent systematic review outlines challenges with changing hand hygiene habits, and proposes that behaviour change is most successful if it is gradual and supported by leadership in a work environment that emphasizes the importance of patient safety [38, 39].

Environmental contamination is an established risk factor for developing a HCAI, with patient rooms serving as a reservoir for multi drug resistant organisms that may infect new and susceptible patients [40, 41]. We observed the cleaning of surfaces as carried out exclusively by hygiene and sanitation staff. The median time for disinfection was 541.5 s, or 9 min 2 s, with a total cost that included products used of $21.9 or 22 cents per action. A longer median time of 14 min 32 s was observed for terminal cleaning, which is done after patients are discharged or transferred. Enhanced patient room disinfection strategies, including those that target terminal cleaning [42] are essential as viruses such as the corona or influenza virus survive on dry surfaces for a few days, while bacteria such as Methicillin-resistant *Staphylococcus aureus* (MRSA) can persist for months [43]. Despite this, few studies assess the costs of environmental cleaning interventions. One initiative, the Researching Effective Approaches to Cleaning in Hospitals (REACH) study, tested an environmental cleaning bundle in 11 Australian hospitals [35]. The bundle was successful in reducing infections and pathogen counts, and the implementation cost of $349,000 Australian dollars (AUD) generated $147,500 in cost savings. Infections prevented from MRSA and Vancomycin-resistant enterococci returned a conservatively estimated net monetary profit of $1.02 million AUD. Costs were obtained for human resources required to implement and receive training in the intervention, but neither regular human resource (time) nor product/material use were assessed. Considering the importance of hospital environmental cleaning, more micro costing analyses of human and product resource costs are required.

Although this pilot study took place prior to the beginning of the pandemic, the basic and additional precautions undertaken by staff were the CBPs with the highest calculated costs. The donning and removing of personal protective equipment (PPE) had a median total cost of $546.8, or 76 cents per action. Twenty percent (20%) of this (16 cents) was attributable to the time it took staff to put on gloves, gowns or masks, while the majority of the cost (80% or 60 cents) represented the materials used per action. For isolation measures, the median total costs were $235.3 for two hours of observation, or $4.1 per action. Over the course of a regular shift of eight hours this would represent a total median cost of $941.3. However, during the study period, no observations of airborne or airborne-contact precautions occurred.

In the current state of the COVID-19 pandemic, PPE is increasingly used for the treatment of all patients, and new equipment such as eye protection (face shield or goggles) is becoming standard practice. This increased use parallels increased costs, as was seen during the Middle East Respiratory Syndrome (MERS-CoV) epidemic. In one hospital in Saudi Arabia with 17 positive cases of MERS, the use of surgical masks increased fivefold and the use of N 95 masks increased tenfold per 1,000 patient days [44]. In the three month period studied, allowing also for the increase in compliance of hand hygiene, this resulted in a $16,400 per month increase in IPC costs. During this past year, this same driving force in the supply chain, coupled with demand in the general public for PPE, has caused the market demand to explode, resulting in global shortages and price increases [45]. In March of 2020, the WHO reported that prices of surgical masks had already increased six fold, N95 respirators had tripled, and surgical gown prices had doubled [46]. Our study contributes new knowledge related to pre-COVID-19 costs which will allow researchers to compare future PPE use and cost increases.

Due to the small number (N = 3) of screening tests done during the one-month study period we were unable to calculate the costs of human or product resources for infectious disease screening. Future work planned by our team will allow for a one-year time frame from which to collect data on screening from medical records.

Overall, the time motion observations using our guide were feasible and acceptable to both the observers and staff being observed. We ensured that the same observer followed a staff member during the course of the study for two hours and 10 min each day. The initial 10 min was not measured, but allowed the observer to adjust to the pace being set by the staff member being observed. These procedures allowed for some dissipation of the Hawthorne effect, a known confounder in observational studies of healthcare practices such as hand washing [47]. The micro-costing data collected in this study were captured with an observational prospective study design, the optimal technique for obtaining accurate cost estimates to inform resource allocation decisions [25].

Our study has several limitations. We were unable to capture two category costs: those of screening and the costs of materials used for the cleaning of small equipment (missing data). We did not include doctors in this pilot project; their higher wages would augment the average human resource cost across the average costs presented here. The pilot test was undertaken between November 28^th^ and December 15^th^; this may not reflect the fluctuation in IPC that may occur due to seasonal variation in diseases. To address these limitations and to test the generalizability of these results, we are undertaking a larger scale study over a one year time horizon using the same time motion guide.

## Conclusions

The cost data retrieved from this study should be of great interest to policy makers, as even by conservative estimates the cost of the CBPs assessed were very low, from 20 cents to $4.1 per action. Our results are relevant not only to stakeholders in Quebec’s healthcare system but in other provinces and countries as well, as they provide arguments with which to make evidence-based decisions of resource allocation that affect the quality and safety of patient care. In this newly heightened context of COVID-19 risk of contagion, research in IPC is increasingly important to prioritize [48]. There is an urgent need to understand that IPC programs are cost-effective. These programs must be acceptable to public officials, administrators, patients, and increasingly, the healthcare workers that are in contact with pathogens on a daily basis. If not, compliance with IPC measures will be low. As well, investments should also demonstrate a cost–benefit, such that the long term repercussions of patient safety are considered. This information will ultimately be useful for the care of the general population.

## Supplementary Information

**Additional file 1.** Algorithm.

**Additional file 2.** Time Motion Guide.

**Additional file 3.** Prices of items used in IPC.

## Data Availability

The datasets used and/or analysed during the current study are available from the corresponding author on reasonable request.

## Abbreviations

BD: Bruno Dubreuil
CBP: Clinical best practice
CDC: Centers for disease control
CISSS: Integrated centre for health and social services
CIUSSS: Integrated university centre for health and social services
COVID-19: Corona Virus 2019
CL: Catherine Larouche
DS: Drissa Sia
ET: Eric Tchouaket
HCAI: Healthcare associated infection
JL: Josiane Létourneau
KK: Kelley Kilpatrick
MRSA: Methicillin-resistant *Staphylococcus aureus*
NP: Natasha Parisien
SB: Sandra Boivin
SR: Stephanie Robins
WHO: World Health Organization
