# Supplementary material for: A pre-pandemic COVID-19 assessment of the costs of prevention and control interventions for healthcare associated infections in medical and surgical wards in Québec
Source: Antimicrob Resist Infect Control. 2021 Oct 21;10:150. doi: 10.1186/s13756-021-01000-y (PMC8529371; doi:10.1186/s13756-021-01000-y)
Supplement: Supplementary file 1 — Additional file 1. Algorithm. [file 13756_2021_1000_MOESM1_ESM.docx]

|  |  |  |  |  |  | Hand Hygiene | Hydroalcoholic solution (HAS) |  |
| --- | --- | --- | --- | --- | --- | --- | --- | --- |
|  |  |  |  |  |  |  | Soap and water |  |
|  |  |  |  |  |  |  |  |  |
|  |  |  |  |  |  | Personal protective equipment (PPE) | Gloves | Disposable |
|  |  |  |  | None |  |  | Mask |  |
|  | Outside, in front of room |  |  | Contact |  |  | Gown | Reusable, Disposable |
|  | Private room (1 bed) |  |  | Enhanced contact |  |  | Eyewear |  |
| Area* | Semi-private room (2 beds) | 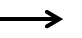 | Additional precautions | Contact-Droplets | 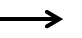 |  | Face Shield |  |
|  | Multi-bed room (>2 beds) |  |  | Droplets |  |  |  |  |
|  | Shared shower or toilet |  |  | Aerial |  | Disinfection  of patient care equipment | Disposable microfibre Disinfecting wipes | Quaternary ammonium, hydrogen peroxide, Chlorine |
|  |  |  |  | Contact-aerial |  |  | Reusable microfibre Disinfecting wipes | Quaternary ammonium, hydrogen peroxide, Chlorine |
|  |  |  |  |  |  |  |  |  |
|  |  |  |  |  |  | Disinfection Hygiene and Cleanliness | Daily |  |
|  |  |  |  |  |  |  | Terminal |  |
|  |  |  |  |  |  |  | Outbreak |  |
|  |  |  |  |  |  |  |  |  |
|  |  |  |  |  |  | Screening | C.*Difficile*, |  |
|  |  |  |  |  |  |  | MRSA |  |
|  |  |  |  |  |  |  | VRE |  |
|  | *not assessed in this study |  |  |  |  |  | CPE (CPGNB) |  |
