# Supplementary material for: A pre-pandemic COVID-19 assessment of the costs of prevention and control interventions for healthcare associated infections in medical and surgical wards in Québec
Source: Antimicrob Resist Infect Control. 2021 Oct 21;10:150. doi: 10.1186/s13756-021-01000-y (PMC8529371; doi:10.1186/s13756-021-01000-y)
Supplement: Supplementary file 2 — Additional file 2. Time Motion Guide. [file 13756_2021_1000_MOESM2_ESM.docx]

Time and Motion Guide Web Platform: paper version

Contents

[Identification: Unit observed 1](#_Toc40046754)

[Healthcare Personnel Identification 6](#_Toc40046755)

[b-Hand Hygiene 7](#_Toc40046756)

[c-Basic care practice / Additional Precautions 8](#_Toc40046757)

[d-Personal Protective Equipment (PPE) 9](#_Toc40046758)

[e-Hygiene and Sanitation 10](#_Toc40046759)

[f-Cleaning of Small Equipment 12](#_Toc40046760)

[g-Screening 13](#_Toc40046761)

NB : *= obligatory field

# Identification: Unit Observed

Identification of Health Care Facility

- site choice * (*hospital*) ➀➁➂➃➄➅

Number of beds in the facility (as per permit) *____________

Number of beds in the unit (as per permit) *_______________

Number of sinks in the corridors of unit*__________________

Hydroalcoholic solution (HAS) dispensers in the unit**(check all that apply)*

- Doors of rooms → if checked:
  - Number of Hydroalcoholic solution (HAS) dispensers at doors of room *________

- Corridor → *if checked*:
  - Number of HAS dispensers in the corridor *______________
- Inside of rooms → *if checked*: indicate kind of room *

| - - Private room (1 bed)   - Semi-private room (2 beds)   - Room with 3 beds   - Room with 4 beds   - Room with 5 beds   - Room with 6 beds   - Other   Other______________________ | Number of HAS dispensers in room *:  **1 2 3 4 5 6 7 8 9 10** |
| --- | --- |

Format of hydroalcoholic solution in unit* *(check all that apply)*

- Foam → *if checked:*
  - Brand of FOAM hydroalcoholic solution in unit*______________________
  - Size (Format):
- 1 litre
- 500 ml
- Compact (Pocket) Size
- Gel/Liquid→ *if checked:*
  - Brand of Gel/Liquid hydroalcoholic solution in unit*____________________
  - Size (Format) of the HAS Gel/Liquid:
- 1 litre
- 500 ml
- Compact (Pocket) Size

Brand of soap used in unit*______________________________________

Total quantity of soap used in unit April 1st to March 31st*______________________

Brand of the 1st hygiene and sanitation product with dosing device (diluent) *___________________

Quantity (in ml) of 1st hygiene and sanitation product with dosing device (diluent) per volume water (e.g. 0.5 ml/4L):_________________________________

Brand of the 2^nd^ hygiene and sanitation product with dosing device (diluent) ________________

Quantity (in ml) of 2^nd^ hygiene and sanitation product with dosing device (diluent) per volume water (e.g. 0.5 ml/4L):_________________________________

Brand of the 1st ready-to-use hygiene and sanitation product *_____________________

Recommended quantity (in mls) of ready-to-use product #1:_______________(ml)

Brand of the 2^nd^ ready-to-use hygiene and sanitation product _____________________

Recommended quantity (in mls) of ready-to-use product # 2:______________(ml)

Brand of disposable disinfecting wipes*________________________________

Total number of boxes of disposable disinfecting wipes used between April 1st and March 31st *

Which of the following excreta management materials do you use? * *(check all that apply)*

|  | Quantity used between April 1 and March 31  * |
| --- | --- |
| - Disposable bedpan → *if checked:* - Reusable bedpan → *if checked:* - Disposable urinal → *if checked:* - Reusable urinal → *if checked:* - Disposable emesis basin/vomit bag holder → *if checked:* - Reusable emesis basin → *if checked:* | 1 2 3 4 5 6 7 8 9 10  1 2 3 4 5 6 7 8 9 10  1 2 3 4 5 6 7 8 9 10  1 2 3 4 5 6 7 8 9 10  1 2 3 4 5 6 7 8 9 10  1 2 3 4 5 6 7 8 9 10 |

Brand of hygienic covers used ________________________________________

Quantity of hygienic urinal covers used between April 1st and March 31^st^ ____________

Quantity of hygienic covers used for emesis basin between April 1st and March 31^st^________________________________________________

Other materials used (list):

________________________________________________________________________________________________________________________________________________

Have you conducted TRAINING SESSIONS in the subject of NIPC between April1st and March 31st?

- No
- Yes → *if checked*:

What is the general profile of the person conducting the training? *(several choices possible*) *

- - Clinical Nurse Specialist in NIPC → *if checked*:
    - how many total training sessions were given by a **clinical nurse specialist** in NIPC between April1st and March 31st * ________________________
  - IPC consultant → *if checked*:
    - how many total training sessions were given by an **IPC consultant** between April1st and March 31st * ________________________
  - Nursing advisor → *if checked*:
    - how many total training sessions were given by a **nursing advisor** in between April1st and March 31^st^? * ________________________
  - Manager → *if checked*:
    - how many total training sessions were given by a **manager** between April1st and March 31^st^? * ___________________
  - Other ___________________

- - Average duration (in minutes) of the training session? *
- 0-15 minutes
- 16-30 minutes
- 31-45 minutes
- 46-60 minutes
- 61 minutes or more

Per session, what is the number of health care professionals who were trained, by profession:

______nurses

______auxiliary nurses

______orderlies

______ hygiene and sanitation staff

______ physicians

Have you conducted INFORMATION SESSIONS in NIPC between April1st and March 31st?

- No
- Yes → *if checked*: how many INFORMATION SESSIONS were held between April1st and March 31st? *____________________
  - Average duration (in minutes) of the training session? *
- 0-15 minutes
- 16-30 minutes
- 31-45 minutes
- 46-60 minutes
- 61 minutes and over

Have you conducted AWARENESS CAMPAIGNS in NIPC between April1st and March 31st?

- No
- Yes → *if checked*:
  - how many AWARENESS CAMPAIGNS were held? *____________
  - Average duration (in days) of the AWARENESS CAMPAIGNS? * ____________________

Would you be able to provide a list of materials used, on average, during these AWARENESS CAMPAIGNS?

______________________________________________________________________________________________________________________________________________________________________________________________________

# Healthcare Personnel Identification

Identification of Health Care Facility (hospital)  * ➀➁➂➃➄➅

Name of unit within hospital **(one choice only)*

- Medicine
- Surgery

Healthcare personnel being observed: Identification *

- Auxiliary Nurse
- Nurse (College Trained)
- Nurse (BSc.)
- Nurse Clinician
- General Practitioner
- Medical Specialist
- Orderly
- Hygiene and sanitation worker

Number of years experience in the current position *

- 0-1 year
- 1-5 years
- 5-10 years
- 10 years and over

Code assigned to person being observed ______________

# b-Hand Hygiene

Hand Hygiene

Moment * Choose the moment that corresponds best to the beginning of the action *(one choice only)*

- Before coming in contact with the patient or their environment
- Before an aseptic procedure
- After handing body fluids or after removal of gloves
- After coming in contact with the patient or their environment
- Other ___________________________________

Products used * *(one choice only)*

- Soap and water
- Hydroalcoholic solution on table (500 ml)
- Hydroalcoholic solution on wall (1L)
- Hydroalcoholic solution pocket size (45-50 ml)
- Other _____________________________________

# c-Basic care practice / Additional Precautions

Additional precautions **(one choice only)*

- Contact
- Droplet
- Droplet - contact
- Airborne
- Airborne - contact
- Protection (inversed)

# d-Personal Protective Equipment (PPE)

Personal protective equipment (PPE)

Action of donning or removing equipment* *(one choice only)*

- Donning
- Removal

PPE used * *(check all that apply)*

- Nitrile gloves
- Vinyl gloves
- Sterile gloves
- Procedural or surgical mask
- N95 Mask
- Disposable gown
- Reusable gown
- Disposable glasses
- Reusable glasses
- Disposable visor
- Reusable visor
- Overshoes
- Other _____________________________________

# e-Hygiene and Sanitation

Hygiene and sanitation

Location where action is being observed **(one choice only)*

- Outside of room *→if checked*  *
- Corridor (hand rail)
- Corridor (hand hygiene station)
- Shower for patients
- Washroom for patients and visitors
- Other_____________________________
- Inside of room*→ if checked* : indicate type of room **(one choice only)*
  - Private room (1 bed)
  - Semi-private room (2 beds)
  - Room with 3 beds
  - Room with 4 beds
  - Room with 5 beds
  - Room with 6 beds
  - Other

Kind of Cleaning **(one choice only)*

- Daily regular
- Daily additional precautions
- Terminal additional precautions *→ if checked* :
  - One step
  - Two steps
  - Three steps
- Terminal discharge /transfer
- Outbreak (high touch surfaces)

| Materials used **(check all that apply)* | |
| --- | --- |
| - Reusable cloths   (washcloths, cotton cloths etc.)   - Microfiber cloths - Disinfectant disposable wipes - Alcohol swabs - Floor buffers - Other ________________________ | Quantity  1 2 3 4 5 6 7 8 9 10  1 2 3 4 5 6 7 8 9 10  1 2 3 4 5 6 7 8 9 10  1 2 3 4 5 6 7 8 9 10  1 2 3 4 5 6 7 8 9 10 |

Products used * *(check all that apply)*

- Quaternary ammonium ready to use
- Quaternary ammonium to dilute
- Hydrogen peroxide ready to use
- Hydrogen peroxide to dilute
- Chlorine solution ready to use
- Chlorine solution to dilute
- Other___________________________

# f-Cleaning of Small Equipment

Cleaning of small equipment

Type of shared health care equipment * *(check all that apply)*

- Multifunction vital signs monitor
- Bassin
- Commode chair
- Wheelchair
- Stretcher
- Blood glucose monitor
- Emesis basin
- Walker
- Positive displacement pump
- Syringe pump
- Pulse oximeter
- Portable bladder scanner
- Stethoscope
- Measuring cup
- Blood pressure monitor
- Thermometer
- I.V. Stand
- Urinal
- Other___________________

| Materials used **(check all that apply)* | |
| --- | --- |
| - Reusable cloths   (washcloths, cotton cloths, etc.)   - Microfiber cloths - Disinfectant disposable wipes - Alcohol swabs - Other ________________________ | Quantity  1 2 3 4 5 6 7 8 9 10  1 2 3 4 5 6 7 8 9 10  1 2 3 4 5 6 7 8 9 10  1 2 3 4 5 6 7 8 9 10 |

Products * *(check all that apply)*

- Quaternary ammonium
- Hydrogen peroxide
- Chlorine solution
- Alcohol
- Other___________________________

# g-Screening

Screening

Screening for

- C. *difficile* (clinical specimen)
- MRSA *→ if checked* :

For MRSA, note site of sample *

- Groin and armpits
- Colostomy
- Nostrils
- Wound(s)
- Tracheostomy
- Urine
- Other ___________________________

Number of samples (swabs) used for MRSA *

1 2 3 4 5 6 7 8 9 10

- VRE→ *if checked* :

For VRE, note site of sample *

- Colostomy
- Wound(s)
- Rectum
- Tracheostomy
- Urine
- Other ___________________________

Number of samples (swabs) used for VRE used*

1 2 3 4 5 6 7 8 9 10

- CPGNB → *if checked* :

For CPGNB note site of sample: *

- Groin and armpits
- Colostomy
- Wound(s)
- Rectum
- Tracheostomy
- Urine
- Other ___________________________

Number of samples (swabs) used for CPGNB *

1 2 3 4 5 6 7 8 9 10
