# Supplementary material for: A pre-pandemic COVID-19 assessment of the costs of prevention and control interventions for healthcare associated infections in medical and surgical wards in Québec
Source: Antimicrob Resist Infect Control. 2021 Oct 21;10:150. doi: 10.1186/s13756-021-01000-y (PMC8529371; doi:10.1186/s13756-021-01000-y)
Supplement: Supplementary file 3 — Additional file 3. Prices of items used in IPC. [file 13756_2021_1000_MOESM3_ESM.docx]

Table 4. Prices of common items used in IPC 2018^*^

| **Products, Materials and Supplies (depreciation)** | **Price** | **Format** |
| --- | --- | --- |
| Washing and disinfecting solutions |  |  |
| Hand Soap | $ 4.45 | 1 liter |
| Hydroalcoholic solution – Wall format | $ 10.81 | 1.3 liters |
| Hydroalcoholic solution – Table format | $ 4.20 | 500 ml |
| Hydroalcoholic solution - Pocket format | $ 1.18 | 59 ml |
| Cleaning Products |  |  |
| Quaternary ammonium | $ 15.15 | 3.8 liter |
| Hydrogen Peroxide - diluted | $ 3.70 | 946 ml |
| Hydrogen Peroxide | $ 13.70 | 3.8 liters |
| Chlorine Solution diluted (1000 ppm) | $ 14.95 | 3.8 liters |
| Screening |  |  |
| Swabs | $ 1.50 | Each |
| Cleaning supplies |  |  |
| Microfibre- reusable 16 x 16 inches (500 washes) | $ 3.90 | Each |
| Microfibre reusable cloths 16X19 inches (300 washes) | $ 0.95 | Each |
| Pre-moistenened wipes with hydrogen peroxide 15 x 18 cm | $ 11.36 | Box of 160 |
| Floor buffers 18 inches (500 washes) | $ 10.75 | Each |
| Mop (300 washes) | $ 10.04 | 24 oz |
| Personal Protective Equipment |  |  |
| Disposable Gown | $.94 | Each |
| Reuseable Gown (100 washes) | $ 8.50 | Each |
| Sterile gloves | $ 8.00 | Box of 100 |
| Nitrile gloves | $ 10.00 | Box of 100 |
| Vinyl gloves | $ 4.35 | Box of 150 |
| Procedural mask | $ 5.84 | Box of 50 |

^*^Prices noted are those from contract bids from Quebec’s Ministry of Health and

Social Services in 2018 Canadian dollars
